# Microglia Don’t Treat All Neurons the Same: The Importance of Neuronal Subtype in Microglia-Neuron Interactions in the Developing Hypothalamus

**DOI:** 10.3389/fncel.2022.867217

**Published:** 2022-04-15

**Authors:** Zuri Ngozi, Jessica L. Bolton

**Affiliations:** Neuroscience Institute, Georgia State University, Atlanta, GA, United States

**Keywords:** microglia, heterogeneity, subpopulation, neuron-specific, microglia-neuron interactions, hypothalamus, PVN, development

## Abstract

Microglia are now well-known as integral regulators of brain development, phagocytosing whole neurons, and pruning weak or excess synapses in order to sculpt and refine immature circuits. However, the importance of neuronal subtype in guiding microglial activity has not received much attention until recently. This perspective will delineate what is known about this topic so far, starting with the developing brain as a whole and then focusing on the developing hypothalamus in particular. There is emerging evidence that subpopulations of microglia treat excitatory and inhibitory neurons differently, and our recent work has shown that even the type of neuropeptide produced by the nearby neurons is important. For example, microglia abutting corticotropin-releasing hormone (CRH)-expressing neurons in the paraventricular nucleus of the hypothalamus (PVN) engulf fewer excitatory synapses than do microglia on the borders of the PVN that are not contacting CRH+ neurons. Potential future directions and technical considerations will be discussed in an effort to catalyze this emerging and exciting area of research. Applications of this research may hold promise in creating more specific therapies that target unique subtypes of microglia-neuron interactions in the atypically developing brain.

## Introduction

Microglia are immunocompetent brain cells of the myeloid lineage that shape neural circuits and respond to CNS injury. Our understanding of microglia has evolved significantly since their discovery. What were originally believed to be passive scavengers, responding primarily to neural injury or infection, are now seen as dynamic mediators of typical neurodevelopment. Adaptations in the technology used to probe microglia have been key in furthering our understanding of these unique macrophages. For example, while historically many researchers homogenized the whole brain to make inferences about microglial function, the more recent advent of two-photon imaging and single-cell RNA-seq technology has granted us insight into region-specific, and even cell-specific, microglial activity. These advances in methodology have already begun to reveal potential complexities in microglia-neuron interactions, in which specific neuronal receptors, neurotransmitters, and neural activity fine-tune microglial function.

In this perspective, we will review existing evidence that microglial subpopulations are neuron-specific, as well as region-specific; present our own recent data to this effect; and discuss ideas for how we can modify approaches to microglial analysis going forward, with this in mind. A focus on neuronal subtype-specific approaches to microglial studies will potentially pave the way for more nuanced, relevant findings in investigations of microglial dysfunction and their contributions to disease. We will devote special attention to the developing hypothalamus, as it is a major hub for specialized neuronal subtypes and thus, neuron-specific microglial activity, and presents important implications for neurodevelopmental, neuropsychiatric, and neurodegenerative diseases.

## Microglial Roles in The Developing Brain

The last two decades of research have uncovered important microglial roles in typical neurodevelopment. Microglial contributions to synaptic refinement have been of particular interest in recent years: following the initial discovery that microglia engulf excess weak synapses in the visual system (Schafer et al., 2012) and hippocampus (Paolicelli et al., 2011), others have shown that these resident macrophages also shape synaptic circuits in the auditory (Milinkeviciute et al., 2019) and somatosensory cortex (Miyamoto et al., 2016). The rise of 2-photon imaging studies has revealed that these macrophages not only contact synaptic elements prior to elimination but also display contact-dependent synaptic formation. For example, Miyamoto et al. (2016) found that microglial process contacts with pyramidal neurons in the developing somatosensory cortex preceded the formation of dendritic filopodia by just 10 min. Such divergences in the decision to remove or generate synapses speak to the vast array of microglial functions and the need for more fine-grained analyses in the developing and aging brain.

Beyond synaptic refinement, microglia also phagocytose neural precursor cells in the developing cerebral cortex and newborn cells in the hippocampus during both typical neurodevelopment and adult neurogenesis, respectively (Cunningham et al., 2013; Diaz-Aparicio et al., 2020). These ongoing roles in neuronal population control and synaptic fine-tuning could explain the overlapping causes of microglial dysfunction in both neurodevelopmental and neurodegenerative diseases (Wright-Jin and Gutmann, 2019; Xu et al., 2020).

Microglia also modulate cognitive function and synaptic plasticity. Indeed, simply adding microglia to hippocampal slices increases LTP generation (Hayashi et al., 2006), and inhibiting CX3CL1/CX3CR1 signaling impairs motor learning in mice, as well as tetanus-induced LTP in the rat spinal cord (Rogers et al., 2011; Bian et al., 2015). More specifically, blocking the microglial proinflammatory cytokine IL-1β prenatally disrupts normal hippocampal development and impairs memory performance later in life, and occluding IL-1β signaling during adulthood impairs hippocampus-dependent associative learning and LTP (Goshen et al., 2007). On the other hand, high levels of IL-1β trigger apoptosis in neural precursor cells, potentially explaining the observed detriments in memory formation following overexpression of the proinflammatory cytokine, above and beyond its normal range (Guadagno et al., 2015) and emphasizing the canonical inverted U-shaped function for the impact of cytokine levels on brain function (Bilbo and Schwarz, 2012).

## Microglia Behave in Region-Specific Ways

Early on, gross analyses of microglial populations revealed that gray matter hosts more microglia than white matter (Lawson et al., 1990). Since then, advancements in imaging technology have gone steps further to uncover changes in both inter- and intra-regional microglial populations. For instance, CA3 microglia are less populous than those in the dentate gyrus and CA1 (Tan et al., 2020), and deeper layers of the cerebellum contain more motile microglial processes than superficial layers (Stowell et al., 2018).

It is possible that the heterogeneity in microglia is functionally correlated with shifts in synaptic turnover. Findings of both temporal and spatial changes in microglial density support this idea. For example, Li et al. (2019) noted an increase in microglia in the inner plexiform layer (IPL) during the perinatal period and the first week of life, corresponding to increased sensory stimulation and synaptic refinement during that period. Additionally, there are denser microglial populations in areas that experience ongoing neurogenesis in adulthood, including the hippocampus and olfactory bulb (Tan et al., 2020).

Beyond microglial density, morphological changes can also be seen across development.

In an analysis of sex, age, and regional differences in microglial morphology, Schwarz et al. (2012) found that embryonic microglia were primarily amoeboid, with fewer ramified processes compared to later developmental time points. By postnatal day (P) 4, sex had a significant effect on microglial phenotype. Specifically, within the parietal cortex, hippocampus, and amygdala, males had more microglia of an “activated” morphology compared to females. Interestingly, these sex differences were reversed by P30, with females instead possessing more “activated” microglia than males, and this difference persisted through P60 (Schwarz et al., 2012).

Microglial gene expression is also spatially and temporally distinct. Whereas the cerebellum has a significant upregulation of cell clearance-associated microglial gene-expression programs, correlated with its high rate of neuronal cell death throughout adulthood, microglia in the striatum, where adult neuronal populations are relatively stable, express more genes associated with homeostatic surveillance functions and mature microglial phenotypes (Ayata et al., 2018). Microglia also tend to shift to more homogenous phenotypes across development and express more regulatory genes in adulthood (Masuda et al., 2019). For example, while embryonic microglia express high levels of lysosome-related genes like *Lamp1* and *Ctsb*, postnatal microglia express more homeostatic genes like *Slc2a5* and *Tmem119*. These age-specific changes in gene expression may mediate the observed activity-dependent shifts in microglial density. If so, further transcriptomic and proteomic analyses may help advance our understanding of region-specific microglial activity in both normal and pathological states.

### Microglial Function in the Developing Hypothalamus

The mammalian hypothalamus hosts wide variability in microglial form and function across normal development and in the diseased brain. During embryonic development, microglia play a role in gliogenesis and oligodendrocyte progenitor cell migration in the hypothalamus (Marsters et al., 2020). Microglia are also necessary for masculinizing the preoptic area and the proper development of male sexual behavior (Lenz et al., 2013). Such sex differences in hypothalamic microglia could explain the sexually dimorphic prevalence of immune-related and affective disorders. Already, microglial dysfunction in the hypothalamus has been linked to metabolic, neurodegenerative, and neuropsychiatric disorders (Lucchina and Depino, 2014; Winkler et al., 2017; Tsyglakova et al., 2019; Sugama and Kakinuma, 2020; Milligan Armstrong et al., 2021). Investigations into the developmental and immunological contributions of microglia could elucidate the underlying causes of sex disparities in the incidence of lupus, Alzheimer’s disease (AD), and autism spectrum disorders (ASD), for example.

Microglia have been shown to use bi-directional communication with specific neuronal subpopulations in multiple subregions of the hypothalamus to mediate food consumption (Valdearcos et al., 2014, 2017; Seong et al., 2019). For example, Valdearcos et al. (2017) showed that microglial inflammatory signaling in the mediobasal hypothalamus modulates caloric intake during obesity. These findings also corresponded with a distinct spatial distribution of microglia expressing Iba1, P2YR12, and/or Tmem119, yet again displaying subregion-specific microglial populations.

Microglia in the developing hypothalamus also play a role in shaping the stress response. Specifically, we have recently shown that early-life adversity (ELA) augments the number and function of excitatory synapses onto stress-sensitive hypothalamic corticotropin-releasing hormone (CRH)-expressing neurons as a result of impaired microglial synaptic pruning in the paraventricular nucleus (PVN; Bolton et al., 2022). ELA profoundly impacts both acute and chronic stress responses in adulthood, but selective chemogenetic activation of microglia with Gq-DREADDs in early life prevents the ELA-induced synaptic excess, as well as the prolonged stress hormone secretion, aberrant behavioral threat responses, and chronic adrenal hypertrophy. These results suggest that microglial function in the developing hypothalamus is important for the sculpting of stress-related circuits and later stress-response behavior, which has implications for vulnerability to stress and later neuropsychiatric disorders.

## Microglia Behave in Neuronal Subtype-Specific Ways

The rise of single-cell RNA-seq work in the past few years has granted us more detailed insight into distinct microglial subpopulations. The work of Hammond et al. (2019) in particular highlights the advantages of using this tool to uncover transcriptional signatures of individual microglia across development. For example, during the 1st week of life, microglia taken from subcortical axon tracts of the forebrain and cerebellum display high expression of *Spp1* (Hammond et al., 2019). Interestingly, these specific “Spp1-high” microglial phenotypes could be found adjacent to microglia that did not express *Spp1*, supporting the idea that distinct microglial subpopulations can be found within the same brain region. *Spp1* is also highly expressed in both Alzheimer’s disease- and glioma-associated microglia, suggesting that this gene may serve a regulatory role in typical neurodevelopment, but contribute to neurodegenerative states, or injury responses in specific pathological conditions (Szulzewsky et al., 2015; Keren-Shaul et al., 2017).

Microglia-vasculature interactions also support the intra-regional heterogeneity of microglial functions. Bisht and colleagues recently characterized capillary-associated microglia, which are distinct from parenchymal microglia in both morphology and abundance (Bisht et al., 2021). What is more, microglia seem to use vascular cues to populate certain brain regions, as supported by age-specific microglial colonization of synapse-rich barrel centers in the somatosensory cortex (Mondo et al., 2020). This distinctive migration is evidently mediated by microglial CX3CR1, suggesting that the gradient of neuronal ligand, CX3CL1, provides directional cues, thus highlighting the role of neurons in guiding microglial maturation.

The inter- and intraregional differences observed in microglia are also due to their interactions with heterogenous neuronal subtypes throughout the central nervous system. Favuzzi and colleagues have recently combined a host of advanced techniques, including single-cell RNA-seq and *in vivo* 2-photon imaging, to investigate distinct microglial subpopulations in the somatosensory cortex (Favuzzi et al., 2021). In addition to noting a significant increase in inhibitory synapses following microglial depletion, they also report subclasses of microglia contacting either predominantly parvalbumin (PV+) inhibitory synapses or primarily excitatory synapses. Remarkably, the investigators found that in the subpopulation that has the most contacts with PV+ interneurons, microglia expressed both *Gabbr1* and *Gabbr2*, indicative of GABA_B_-receptivity, and eliminating GABA_B1_ receptors specifically in microglia augmented inhibitory connectivity. Meanwhile, the subpopulation of microglia that primarily contacts excitatory synapses is less likely to express GABA receptors. This distinction could help elucidate the pathological causes of altered excitatory and inhibitory circuits in the brain.

Recent work has broadened our understanding of neuronal receptor-specific aspects of microglial heterogeneity. In 2014, researchers found that neuronal NMDAR binding induces microglial process outgrowth *via* increased ATP release (Dissing-Olesen et al., 2014). Since then, Eyo et al. (2018) have revealed temporally distinct roles of the neuronal NMDAR subunit GluN2A in guiding microglia-neuron contact during synaptic plasticity. Correlating with the developmental switch from predominantly GluN2B expression to GluN2A expression, NMDAR activation elicited microglial process outgrowth in tissue from hippocampal CA1 of P30 animals, whereas NMDA activation in tissue from P7 pups failed to induce a similar microglial response. This developmental shift may also underlie the increased synaptic pruning in areas that continue to experience rich synaptic turnover during adulthood.

### Neuron-Microglia Crosstalk in the Developing Hypothalamus

While most cell-specific research on microglia focuses on cortical regions and the hippocampus due to ease of access/manipulation and contributions to LTP, respectively, the range of neuronal subtype diversity in the hypothalamus makes it an especially promising region for investigation of microglial heterogeneity. Long-distance hormone release has already been shown to regulate microglial cytokine expression in the hypothalamus. For example, leptin-exposed hypothalamic microglia express IL-1β and TNF-α (Gao et al., 2014). Moreover, systemic increases in leptin release due to high-fat diet exposure leads to the recruitment of microglia into the arcuate nucleus. If microglia are able to engage in this type of distant communication, it would not be implausible to propose that these macrophages also participate in local, hormone- and neurotransmitter-specific activity within the hypothalamus.

**Figure 1:**
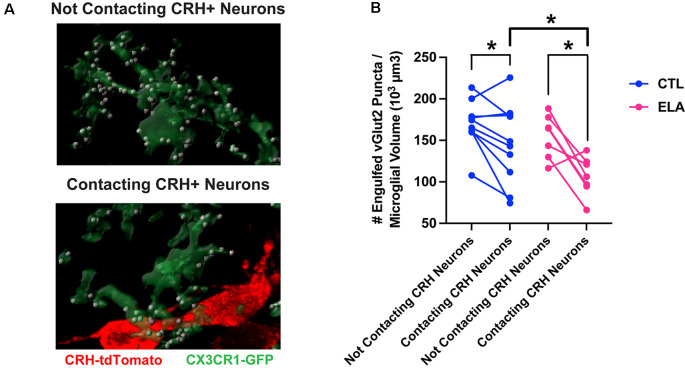
Microglial subpopulations in the paraventricular nucleus of the hypothalamus (PVN) differ in their synaptic pruning levels based on CRH+ neuron contact. **(A)** Representative confocal images of 3D-reconstructed microglia (CX3CR1-GFP+; green) and their engulfed vGlut2+ synaptic puncta (white) in the subpopulations that contact CRH+ neurons (tdTomato+; red; bottom) and those that do not (top) in the PVN of a postnatal day (P) 8 male ELA mouse. **(B)** Only microglia in the PVN that are directly abutting CRH+ neurons are inhibited by ELA and prune fewer synapses compared to controls (unpaired *t*-test with Welch’s correction; CTL vs. ELA: contacting CRH+ neurons, *t*_13.9_ = 2.22, *p* = 0.04). Furthermore, microglia contacting CRH+ neurons engulf fewer vGlut2+ synaptic puncta compared to microglia not contacting CRH+ neurons in both CTL and ELA mice at P8 (paired *t*-tests; CTL: contacting vs. not, *t*_9_ = 2.46, *p* = 0.04; ELA: contacting vs. not, *t*_6_ = 3.54, *p* = 0.01). Data are mean ± SEM. *p < 0.05. Adapted from Bolton et al. (2022).

Indeed, Rosin et al. (2021) have recently discovered a subpopulation of embryonic hypothalamic microglia that interact with neural stem cells lining the third ventricle and are responsive to maternal stress. We have also reported the presence of microglial subpopulations in the PVN in terms of their response to ELA and their subsequent level of synaptic pruning (Bolton et al., 2022). Specifically, only microglia in the PVN that are directly abutting CRH+ neurons are inhibited by ELA and prune fewer synapses (Figure 1). This suggests a neuron-to-glia information flow initiated by ELA, in which CRH+ neurons are altered by early-life experiences, as we have previously shown (Singh-Taylor et al., 2015; Bolton et al., 2020; Short et al., 2021), then signal differentially to microglia, potentially *via* microglial CRH and/or glucocorticoid receptors, to inhibit their synapse engulfment. With microglia known to express such neuropeptide and hormone receptors (Zhang et al., 2014), in addition to neurotransmitter receptors, it’s possible that they are “listening in” on neuron-to-neuron communication, which further shapes their functionality in a neuronal subtype-specific manner.

## Considerations for Studying Microglia in Neuron-Specific Ways

As discussed above, the field has already made significant progress in advancing techniques that have enabled the discovery of intra-regional heterogeneity and neuron-specific subsets of microglia. However, moving forward with these new discoveries in mind requires that we continue improving and advancing the techniques we use to investigate microglia.

**Figure 2:**
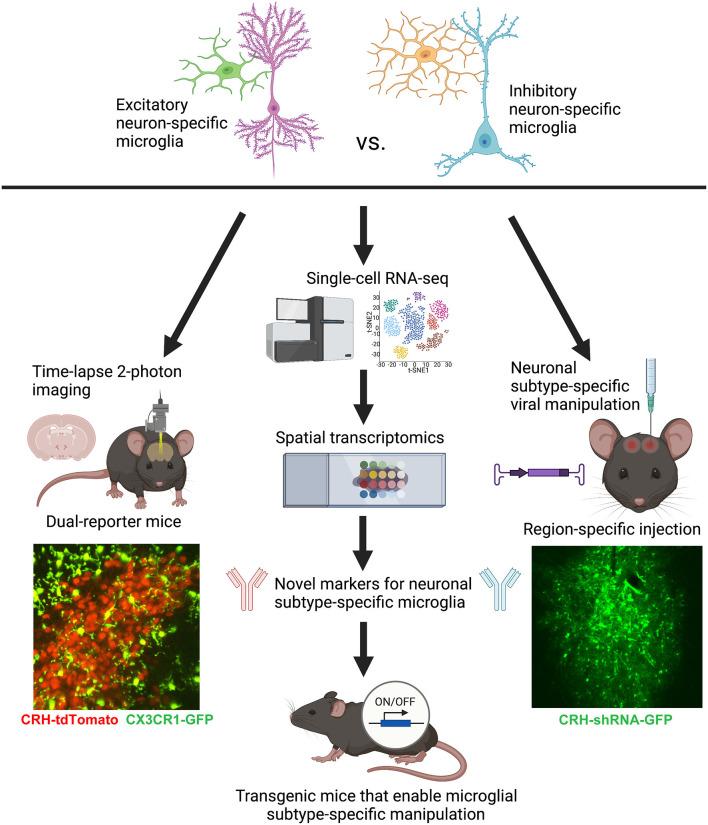
Conceptual figure displaying the proposed technical considerations and future studies that would advance the field of microglial biology by enabling the study of neuronal subtype-specific microglia (e.g., excitatory- vs. inhibitory-specific). Lower left: representative still-frame of a 2-photon time-lapse video recorded from an acute slice of the PVN from a P8 CRH-tdTomato+/–; CX3CR1-GFP+/– mouse. Adapted from Bolton et al. (2022). Lower right: representative image of a lentiviral injection of CRH-shRNA (labeled with GFP) into the central nucleus of the amygdala. Adapted from Bolton et al. (2018). Figure created with BioRender.com.

Considering the inter- and intra-regional heterogeneity of microglial populations in the brain, it is no longer very useful to homogenize the whole brain to isolate microglia and assess gene expression. Punching specific brain regions and performing RNA-seq or scRNA-seq is more appropriate, but still lacks spatial information or any clues as to the neuronal subtypes directly contacted by each microglia. Thus, it is our recommendation that the field rely more heavily on *in situ* analyses, such as spatial transcriptomics and/or spatial proteomics, and highly multiplexed fluorescent *in situ* hybridization and immunohistochemistry. For example, scRNA-seq studies that identify subpopulations of microglia within a brain region can be followed up with MERFISH (Multiplexed Error Robust Fluorescence in situ Hybridization; e.g., Favuzzi et al., 2021) in order to determine *where* exactly these subpopulations are localized in the tissue and whether they contact specific neuronal subtypes and/or the vasculature, potentially providing the underlying reason for the observed heterogeneity in gene expression (Figure 2). Metabolomics and epigenomic analyses, which will likely soon have spatially resolved counterparts, will also prove useful to complement transcriptomic and proteomic analyses for deciphering microglia-neuron crosstalk in defined brain regions (e.g., Ayata et al., 2018; Diop et al., 2018; Chucair-Elliott et al., 2020).

Functional studies employing 2-photon time-lapse imaging of microglia, not just in isolation, but interacting with specific neuronal subtypes in dual-reporter mice (Figure 2) will be important complementary studies to gene/protein expression. For example, we recently imaged microglial process dynamics in CRH-tdTomato+/–; CX3CR1-GFP+/– mice (see Figure 2) to understand how microglia abutting CRH+ neurons in the PVN were impacted by ELA. It turned out that these microglia, which were also impaired in terms of synaptic pruning, had significantly diminished process dynamics—a potential cause of the deficits in synapse engulfment (Bolton et al., 2022). It will be important moving forward to image microglial subpopulations in contact with specific neuronal subtypes in brain regions of interest for the system and question at hand.

Unfortunately, it is currently very difficult to manipulate microglial subpopulations in a region-specific manner due to the relative ineffectiveness of viral approaches in transfecting microglia (Maes et al., 2019). The best available approach that approximates the manipulation of specific microglia is using viral transfection of specific neuronal subtypes, targeted into select brain regions, in order to manipulate the neuronal partners for microglial subpopulations of interest (e.g., *via* neuronal ligands that are bound by microglia, such as CX3CL1, C1q, etc.; see Figure 2; Bolton et al., 2018; Werneburg et al., 2020). However, there is hope that sc-RNA-seq combined with spatial transcriptomics may allow us to identify novel genetic markers for microglial subpopulations that will eventually make these specific microglia, rather than all microglia (such as in current mouse lines utilizing CX3CR1; P2RY12; Tmem119, etc.; Bennett et al., 2016), genetically tractable using the Cre-lox system (Figure 2). The continued development of technologies such as these will prove invaluable for advancing the field of microglial biology in the coming years.

## Discussion

Our understanding of microglia and other heterogenous brain cells have become increasingly dynamic with modern technological and methodological advancements. In recent years, studies that have revealed microglial contributions to synaptic refinement, neural circuit formation, and neuronal population regulation have broadened our perceptions of these brain-resident macrophages. One could argue that our constantly evolving approaches to investigating microglia are due to our gradual transition out of “one-size-fits-all” ideas concerning their function. Indeed, with every step we collectively move towards advancing techniques in microglia research, we get closer to unveiling fascinating cell-specific interplays between microglia and their unique microenvironments. And while techniques like single-cell RNA-seq and spatial transcriptomics are certainly driving these important discoveries, we must continue to seek novel technologies to uncover distinct subtypes of microglial function in both healthy and diseased states.

## Data Availability Statement

The raw data supporting the conclusions of this article will be made available by the authors, without undue reservation.

## Ethics Statement

The animal study was reviewed and approved by Institutional Animal Care and Use Committee (IACUC), University of California—Irvine.

## Author Contributions

JB and ZN wrote and edited the article. JB performed the experiments, analyzed the data, and created the figures for the data included in this Perspective article. All authors contributed to the article and approved the submitted version.

## Funding

This work was supported by National Institute of Mental Health (NIMH) grant K99/R00 MH120327 (JB), and the open access publication fees will be paid from this grant. The funder did not have any role in the experiments reported or the article as written.

## Conflict of Interest

The authors declare that the research was conducted in the absence of any commercial or financial relationships that could be construed as a potential conflict of interest.

## Publisher’s Note

All claims expressed in this article are solely those of the authors and do not necessarily represent those of their affiliated organizations, or those of the publisher, the editors and the reviewers. Any product that may be evaluated in this article, or claim that may be made by its manufacturer, is not guaranteed or endorsed by the publisher.

## References

[B1] Ayata P. Badimon A. Strasburger H. J. Duff M. K. Montgomery S. E. Loh Y.-H. E.. (2018). Epigenetic regulation of brain region-specific microglia clearance activity. Nat. Neurosci. 21, 1049–1060. 10.1038/s41593-018-0192-3 30038282 PMC6090564

[B2] Bennett M. L. Bennett F. C. Liddelow S. A. Ajami B. Zamanian J. L. Fernhoff N. B.. (2016). New tools for studying microglia in the mouse and human CNS. Proc. Natl. Acad. Sci. U S A 113, E1738–1746. 10.1073/pnas.1525528113 26884166 PMC4812770

[B3] Bian C. Zhao Z. Q. Zhang Y. Q. Lü N. (2015). Involvement of CX3CL1/CX3CR1 signaling in spinal long term potentiation. PLoS One 10:e0118842. 10.1371/journal.pone.0118842 25768734 PMC4358970

[B4] Bilbo S. D. Schwarz J. M. (2012). The immune system and developmental programming of brain and behavior. Front. Neuroendocrinol. 33, 267–286. 10.1016/j.yfrne.2012.08.006 22982535 PMC3484177

[B5] Bisht K. Okojie K. A. Sharma K. Lentferink D. H. Sun Y. Y. Chen H. R.. (2021). Capillary-associated microglia regulate vascular structure and function through PANX1–P2RY12 coupling in mice. Nat. Commun. 12:5289. 10.1038/s41467-021-25590-8 34489419 PMC8421455

[B6] Bolton J. L. Molet J. Regev L. Chen Y. Rismanchi N. Haddad E.. (2018). Anhedonia following early-life adversity involves aberrant interaction of reward and anxiety circuits and is reversed by partial silencing of amygdala corticotropin-releasing hormone gene. Biol. Psychiatry 83, 137–147. 10.1016/j.biopsych.2017.08.023 29033027 PMC5723546

[B7] Bolton J. L. Schulmann A. Garcia-Curran M. M. Regev L. Chen Y. Kamei N.. (2020). Unexpected transcriptional programs contribute to hippocampal memory deficits and neuronal stunting after early-life adversity. Cell Rep. 33:108511. 10.1016/j.celrep.2020.108511 33326786 PMC7817243

[B8] Bolton J. L. Short A. K. Othy S. Kooiker C. L. Shao M. Gunn B. G.. (2022). Early stress-induced impaired microglial pruning of excitatory synapses on immature CRH-expressing neurons provokes aberrant adult stress responses. Cell Rep. 38:110600. 10.1016/j.celrep.2022.110600 35354026 PMC9014810

[B9] Chucair-Elliott A. J. Ocañas S. R. Stanford D. R. Ansere V. A. Buettner K. B. Porter H.. (2020). Inducible cell-specific mouse models for paired epigenetic and transcriptomic studies of microglia and astroglia. Commun. Biol. 3:693. 10.1038/s42003-020-01418-x 33214681 PMC7678837

[B10] Cunningham C. L. Martínez-Cerdeño V. Noctor S. C. (2013). Microglia regulate the number of neural precursor cells in the developing cerebral cortex. J. Neurosci. 33, 4216–4233. 10.1523/JNEUROSCI.3441-12.2013 23467340 PMC3711552

[B11] Diaz-Aparicio I. Paris I. Sierra-Torre V. Plaza-Zabala A. Rodríguez-Iglesias N. Márquez-Ropero M.. (2020). Microglia actively remodel adult hippocampal neurogenesis through the phagocytosis secretome. J. Neurosci. 40, 1453–1482. 10.1523/JNEUROSCI.0993-19.2019 31896673 PMC7044727

[B12] Diop F. Vial T. Ferraris P. Wichit S. Bengue M. Hamel R.. (2018). Zika virus infection modulates the metabolomic profile of microglial cells. PLoS One 13:e0206093. 10.1371/journal.pone.0206093 30359409 PMC6201926

[B13] Dissing-Olesen L. LeDue J. M. Rungta R. L. Hefendehl J. K. Choi H. B. MacVicar B. A. (2014). Activation of neuronal NMDA receptors triggers transient ATP-mediated microglial process outgrowth. J. Neurosci. 34, 10511–10527. 10.1523/JNEUROSCI.0405-14.2014 25100586 PMC6802598

[B14] Eyo U. B. Bispo A. Liu J. Sabu S. Wu R. Dibona V. L.. (2018). The GluN2A subunit regulates neuronal NMDA receptor-induced microglia-neuron physical interactions. Sci. Rep. 8:828. 10.1038/s41598-018-19205-4 29339791 PMC5770428

[B15] Favuzzi E. Huang S. Saldi G. A. Binan L. Ibrahim L. A. Fernández-Otero M.. (2021). GABA-receptive microglia selectively sculpt developing inhibitory circuits. Cell 184, 4048–4063.e32. 10.1016/j.cell.2021.06.018 34233165 PMC9122259

[B16] Gao Y. Ottaway N. Schriever S. C. Legutko B. García-Cáceres C. de la Fuente E.. (2014). Hormones and diet, but not body weight, control hypothalamic microglial activity. GLIA 62, 17–25. 10.1002/glia.22580 24166765 PMC4213950

[B17] Goshen I. Kreisel T. Ounallah-Saad H. Renbaum P. Zalzstein Y. Ben-Hur T.. (2007). A dual role for interleukin-1 in hippocampal-dependent memory processes. Psychoneuroendocrinology 32, 1106–1115. 10.1016/j.psyneuen.2007.09.004 17976923

[B18] Guadagno J. Swan P. Shaikh R. Cregan S. P. (2015). Microglia-derived IL-1β triggers p53-mediated cell cycle arrest and apoptosis in neural precursor cells. Cell Death Dis. 6:e1779. 10.1038/cddis.2015.151 26043079 PMC4669832

[B19] Hammond T. R. Dufort C. Dissing-Olesen L. Giera S. Young A. Wysoker A.. (2019). Single-cell RNA sequencing of microglia throughout the mouse lifespan and in the injured brain reveals complex cell-state changes. Immunity 50, 253–271.e6. 10.1016/j.immuni.2018.11.004 30471926 PMC6655561

[B20] Hayashi Y. Tomimatsu Y. Suzuki H. Yamada J. Wu Z. Yao H.. (2006). The intra-arterial injection of microglia protects hippocampal CA1 neurons against global ischemia-induced functional deficits in rats. Neuroscience 142, 87–96. 10.1016/j.neuroscience.2006.06.003 16844302

[B21] Keren-Shaul H. Spinrad A. Weiner A. Matcovitch-Natan O. Dvir-Szternfeld R. Ulland T. K.. (2017). A unique microglia type associated with restricting development of Alzheimer’s disease. Cell 169, 1276–1290.e17. 10.1016/j.cell.2017.05.018 28602351

[B22] Lawson L. J. Perry V. H. Dri P. Gordon S. (1990). Heterogeneity in the distribution and morphology of microglia in the normal adult mouse brain. Neuroscience 39, 151–170. 10.1016/0306-4522(90)90229-w 2089275

[B23] Lenz K. M. Nugent B. M. Haliyur R. McCarthy M. M. (2013). Microglia are essential to masculinization of brain and behavior. J. Neurosci. 33, 2761–2772. 10.1523/JNEUROSCI.1268-12.2013 23407936 PMC3727162

[B60] Li F. Jiang D. Samuel M. A. (2019). Microglia in the developing retina. Neural Dev. 14:12. 10.1186/s13064-019-0137-x 31888774 PMC6938006

[B24] Lucchina L. Depino A. M. (2014). Altered peripheral and central inflammatory responses in a mouse model of autism. Autism Res. 7, 273–289. 10.1002/aur.1338 24124122

[B25] Maes M. E. Colombo G. Schulz R. Siegert S. (2019). Targeting microglia with lentivirus and AAV: recent advances and remaining challenges. Neurosci. Lett. 707:134310. 10.1016/j.neulet.2019.134310 31158432 PMC6734419

[B26] Marsters C. M. Nesan D. Far R. Klenin N. Pittman Q. J. Kurrasch D. M. (2020). Embryonic microglia influence developing hypothalamic glial populations. J. Neuroinflammation 17:146. 10.1186/s12974-020-01811-7 32375817 PMC7201702

[B27] Masuda T. Sankowski R. Staszewski O. Böttcher C. Amann L. Sagar.. (2019). Spatial and temporal heterogeneity of mouse and human microglia at single-cell resolution. Nature 566, 388–392. 10.1038/s41586-019-0924-x 30760929

[B28] Milinkeviciute G. Henningfield C. M. Muniak M. A. Chokr S. M. Green K. N. Cramer K. S. (2019). Microglia regulate pruning of specialized synapses in the auditory brainstem. Front. Neural Circuits 13:55. 10.3389/fncir.2019.00055 31555101 PMC6722190

[B29] Milligan Armstrong A. Porter T. Quek H. White A. Haynes J. Jackaman C.. (2021). Chronic stress and Alzheimer’s disease: the interplay between the hypothalamic-pituitary-adrenal axis, genetics and microglia. Biol. Rev. Camb. Philos. Soc. 96, 2209–2228. 10.1111/brv.12750 34159699

[B30] Miyamoto A. Wake H. Ishikawa A. W. Eto K. Shibata K. Murakoshi H.. (2016). Microglia contact induces synapse formation in developing somatosensory cortex. Nat. Commun. 7:12540. 10.1038/ncomms12540 27558646 PMC5007295

[B31] Mondo E. Becker S. C. Kautzman A. G. Schifferer M. Baer C. E. Chen J.. (2020). A developmental analysis of juxtavascular microglia dynamics and interactions with the vasculature. J. Neurosci. 40, 6503–6521. 10.1523/JNEUROSCI.3006-19.2020 32661024 PMC7486666

[B32] Paolicelli R. C. Bolasco G. Pagani F. Maggi L. Scianni M. Panzanelli P.. (2011). Synaptic pruning by microglia is necessary for normal brain development. Science 333, 1456–1458. 10.1126/science.1202529 21778362

[B33] Rogers J. T. Morganti J. M. Bachstetter A. D. Hudson C. E. Peters M. M. Grimmig B. A.. (2011). CX3CR1 deficiency leads to impairment of hippocampal cognitive function and synaptic plasticity. J. Neurosci. 31, 16241–16250. 10.1523/JNEUROSCI.3667-11.2011 22072675 PMC3236509

[B34] Rosin J. M. Sinha S. Biernaskie J. Kurrasch D. M. (2021). A subpopulation of embryonic microglia respond to maternal stress and influence nearby neural progenitors. Dev. Cell 56, 1326–1345.e6. 10.1016/j.devcel.2021.03.018 33887203

[B35] Schafer D. P. Lehrman E. K. Kautzman A. G. Koyama R. Mardinly A. R. Yamasaki R.. (2012). Microglia sculpt postnatal neural circuits in an activity and complement-dependent manner. Neuron 74, 691–705. 10.1016/j.neuron.2012.03.026 22632727 PMC3528177

[B36] Schwarz J. M. Sholar P. W. Bilbo S. D. (2012). Sex differences in microglial colonization of the developing rat brain. J. Neurochem. 120, 948–963. 10.1111/j.1471-4159.2011.07630.x 22182318 PMC3296888

[B37] Seong J. Kang J. Y. Sun J. S. Kim K. W. (2019). Hypothalamic inflammation and obesity: a mechanistic review. Arch. Pharm. Res. 42, 383–392. 10.1007/s12272-019-01138-9 30835074

[B38] Short A. K. Thai C. W. Chen Y. Kamei N. Pham A. L. Birnie M. T.. (2021). Single-cell transcriptional changes in hypothalamic corticotropin-releasing factor expressing neurons after early-life adversity inform enduring alterations in vulnerabilities to stress. Biol. Psychiatry Global Open Sci. 10.1016/j.bpsgos.2021.12.006. [Online ahead of print]. PMC9874075 36712559

[B39] Singh-Taylor A. Korosi A. Molet J. Gunn B. G. Baram T. Z. (2015). Synaptic rewiring of stress-sensitive neurons by early-life experience: a mechanism for resilience? Neurobiol. Stress 1, 109–115. 10.1016/j.ynstr.2014.10.007 25530985 PMC4267062

[B40] Stowell R. D. Wong E. L. Batchelor H. N. Mendes M. S. Lamantia C. E. Whitelaw B. S.. (2018). Cerebellar microglia are dynamically unique and survey Purkinje neurons *in vivo*. Dev. Neurobiol. 78, 627–644. 10.1002/dneu.22572 29285893 PMC6544048

[B41] Sugama S. Kakinuma Y. (2020). Stress and brain immunity: microglial homeostasis through hypothalamus-pituitary-adrenal gland axis and sympathetic nervous system. Brain Behav. Immun. Health 7:100111. 10.1016/j.bbih.2020.100111 34589871 PMC8474505

[B42] Szulzewsky F. Pelz A. Feng X. Synowitz M. Markovic D. Langmann T.. (2015). Glioma-associated microglia/macrophages display an expression profile different from M1 and M2 polarization and highly express Gpnmb and Spp1. PLoS One 10:e0116644. 10.1371/journal.pone.0116644 25658639 PMC4320099

[B43] Tan Y. L. Yuan Y. Tian L. (2020). Microglial regional heterogeneity and its role in the brain. Mol. Psychiatry 25, 351–367. 10.1038/s41380-019-0609-8 31772305 PMC6974435

[B44] Tsyglakova M. McDaniel D. Hodes G. E. (2019). Immune mechanisms of stress susceptibility and resilience: lessons from animal models. Front. Neuroendocrinol. 54:100771. 10.1016/j.yfrne.2019.100771 31325456

[B45] Valdearcos M. Douglass J. D. Robblee M. M. Dorfman M. D. Stifler D. R. Bennett M. L.. (2017). Microglial inflammatory signaling orchestrates the hypothalamic immune response to dietary excess and mediates obesity susceptibility. Cell Metab. 26, 185–197.e3. 10.1016/j.cmet.2017.05.015 28683286 PMC5569901

[B46] Valdearcos M. Robblee M. M. Benjamin D. I. Nomura D. K. Xu A. W. Koliwad S. K. (2014). Microglia dictate the impact of saturated fat consumption on hypothalamic inflammation and neuronal function. Cell Rep. 9, 2124–2138. 10.1016/j.celrep.2014.11.018 25497089 PMC4617309

[B47] Werneburg S. Jung J. Kunjamma R. B. Ha S. K. Luciano N. J. Willis C. M.. (2020). Targeted complement inhibition at synapses prevents microglial synaptic engulfment and synapse loss in demyelinating disease. Immunity 52, 167–182.e7. 10.1016/j.immuni.2019.12.004 31883839 PMC6996144

[B48] Winkler Z. Kuti D. Ferenczi S. Gulyás K. Polyák Á. Kovács K. J. (2017). Impaired microglia fractalkine signaling affects stress reaction and coping style in mice. Behav. Brain Res. 334, 119–128. 10.1016/j.bbr.2017.07.023 28736330

[B49] Wright-Jin E. C. Gutmann D. H. (2019). Microglia as dynamic cellular mediators of brain function. Trends Mol. Med. 25, 967–979. 10.1016/j.molmed.2019.08.013 31597593 PMC6829057

[B50] Xu Z. X. Kim G. H. Tan J. W. Riso A. E. Sun Y. Xu E. Y.. (2020). Elevated protein synthesis in microglia causes autism-like synaptic and behavioral aberrations. Nat. Commun. 11:1797. 10.1038/s41467-020-15530-3 32286273 PMC7156673

[B51] Zhang Y. Chen K. Sloan S. A. Bennett M. L. Scholze A. R. O’Keeffe S.. (2014). An RNA-sequencing transcriptome and splicing database of glia, neurons and vascular cells of the cerebral cortex. J. Neurosci. 34, 11929–11947. 10.1523/JNEUROSCI.1860-14.2014 25186741 PMC4152602

